# Cardiometabolic syndrome and associated factors among Ethiopian public servants, Addis Ababa, Ethiopia

**DOI:** 10.1038/s41598-021-99913-6

**Published:** 2021-10-19

**Authors:** Zeleke Geto, Feyissa Challa, Tadesse Lejisa, Tigist Getahun, Meron Sileshi, Bikila Nagasa, Yosef Tolcha, Yeabkal Daniel, Misrak Getnet, Meseret Derbew Molla, Maria Degef, Abebe Bekele, Daniel Seifu

**Affiliations:** 1 Department of Biomedical Science, College of Medicine and Health Sciences, Wollo University, Wollo, Dessie, Ethiopia; 2 National Reference Laboratory for Clinical Chemistry, Ethiopian Public Health Institute, Addis Ababa, Ethiopia; 3 Health System and Reproductive Health Research Directorate, Ethiopian Public Health Institute, Addis Ababa, Ethiopia; 4 Department of Biochemistry, College of Health Sciences, University of Gondar, Gondor, Ethiopia; 5 Department of Biochemistry, College of Health Sciences, Addis Ababa University, Addis Ababa, Ethiopia; 6 Division of Basic Sciences, Department of Biochemistry, University of Global Health Equity, Kigali, Rwanda

**Keywords:** Biochemistry, Cardiology, Diseases, Endocrinology, Risk factors

## Abstract

Non-communicable diseases (NCDs) are increasingly becoming the global cause of premature death encompassing cardiovascular diseases (CVDs), cancer, respiratory diseases and diabetes mellitus. However, cardiometabolic risk factors in the general population, especially among the high-risk groups have rarely been assessed in Ethiopia. The study aimed to assess the prevalence of metabolic syndrome, its components and associated factors among staff in the Ethiopian Public Health Institute (EPHI). An institutional-based cross-section study was conducted from March to June 2018 among EPHI staff members. A total of 450 study participants were involved in the study, and the World Health Organization NCD STEPS survey instrument version 3.1 was used for the assessment. The biochemical parameters were analyzed by using COBAS 6000 analyzer. Statistical package for the social science (SPSS) version 20 was used for data analysis. Both bivariate and multivariate logistic regression analyses were used to identify associated risk factors. p value < 0.05 was considered for statistical significance. The overall prevalence of metabolic syndrome was 27.6% and 16.7% according to IDF and NCEP criteria respectively, with males having greater prevalence than females (35.8% vs 19.4%). Central obesity, low high-density lipoprotein (HDL) and hypertension had a prevalence of 80.2%, 41.3%, and 23.6%, respectively. In multivariate analysis increasing age and having a higher body mass index (25–29.9) were significantly associated with metabolic syndromes. The magnitude of metabolic syndrome was relatively high among public employees. Preventive intervention measures should be designed on the modification of lifestyle, nutrition and physical activities, and early screening for early identification of cardiometabolic risks factors should be practised to reduce the risk of developing cardiovascular diseases.

## Introduction

According to World Health Origination (WHO), non-communicable diseases (NCDs) are increasingly becoming the leading cause of morbidity and mortality involving every country worldwide^1^. NCDs, such as cardiovascular diseases (CVDs), different types of cancers, diabetes, and chronic respiratory diseases are the global leading causes of deaths which are responsible for 70% of all deaths worldwide^2^. From 36 million annual NCD deaths WHO report, CVDs stand the first place and accounts for 17.5 million followed by cancers (8.2 million), respiratory diseases (4.0 million) and diabetes mellitus (1.5 million)^3^.

NCDs shared common and key modifiable behavioural risk factors like unhealthy diet, lack of physical activity, use of alcohol, and tobacco; all that in turn leads to overweight/obesity, raised blood pressure, raised cholesterol, raised blood glucose and finally chronic diseases^2^. These risk factors for cardiometabolic syndrome have shown clustering and synergizing effects through time and then associated with a higher prevalence of NCDs, primarily CVDs and type 2 diabetes-related mortality^4–7^ The rise in the magnitude of cardiometabolic risks factors, such as obesity, hyperglycemia, hypertension, and dyslipidemia; CVDs become the leading causes of premature mortality^8^. Modifiable risk factors like high rates of smoking, alcohol consumption, poor diet and limited physical activities have been commonly practised and believed to be major risk factors of getting cardiometabolic diseases^8,9^. While Ethiopia has significant progress in reducing the burden of infectious diseases, but there is little known about cardiometabolic diseases, their prevalence, and associated factors among government employees in Ethiopia. Therefore, our study aimed at evaluating the prevalence of cardiometabolic diseases and their associated factors among staff in EPHI.

## Materials and methods

### Study design and setting

An institutional-based cross-sectional study was conducted using the WHO STEPwise survey tool from March 2018 to June 2018 by experienced and well-trained data collectors. The survey involves three steps assessing the socio-demographic, behavioural characteristics, physical and clinical measurement, and biochemical measurement. The study includes all staff members of EPHI with excluding pregnant women during data collection.

### Data collections

#### Demographic and lifestyle factors

Demographic, socioeconomic status, smoking status, alcohol consumption, physical activities status, fruit and vegetable consumption, history of raised blood pressure or medication for blood pressure was collected using standard personal digital assistants (PDAs) and transferred to the central server using an internet file streaming systems (IFSS).

Height and body mass were measured using calibrated scale and body mass index (BMI: kg/m^2^)^10^ were calculated. Waist circumference was measured in centimetres at the narrowest point between the lower costal border and the iliac crest using a tape meter and waist-to-height ratio (WHtR) calculated from waist circumference and height^11^.

Blood pressure measurements were taken three times at the midpoint of the left arm after participants rest for at least five minutes or 30 min for those who took hot drinks using a Boso-Medicus Uno instrument (Boso, Germany) and average were taken. Physical activity was categorized into vigorous, moderate and sedentary (low) activity. A vigorous-intensity activity was defined as any activity that causes a large increase in breathing or heart rate if continued for at least 10 min and 3 days per week (e.g. running, carrying or lifting heavy loads, digging or construction work). Moderate-intensity activity was defined as any activity that causes a small increase in breathing or heart rate if continued for at least 10 min (brisk walking or carrying light loads). Moderate can also define by meeting any of the following criteria: three or more days of vigorous-intensity activity of at least 20 min per day; or five or more days of moderate-intensity activity or walking for at least 30 min per day. Physical activity related to work, transportation and leisure time was assessed in terms of minutes that caused them to breathless or feel palpitation. Low-level physical activity involves a person not meeting any of the above-mentioned criteria for the moderate- or high-level categories^12^.

Fruit and vegetable consumption was assessed by asking participants the number of days and serving they ate fruits and vegetables in a typical week. According to WHO guidelines, servings were measured by showing the study participants show cards showing that one standard serving size equals 80 g.

Alcohol consumption and smoking status were assessed based on a Yes/No response. Participants who consume any amount of alcohol in the past 30 days were considered alcohol consumers^13^. Khat (Catha Edulis Forsk) is a green leaf that has a stimulant effect and is common in East Africa and the Middle East. The study participants were assesses based on the current chewer, previous chewer, and never chewer.

### Biochemical analysis

Blood samples were collected from study participants after overnight fasting for 8–10 h. The collected specimen was allowed to clot for 30 min at room temperature and then centrifuged at 5000 rpm for 5 min. Serum separated from whole blood transferred into 2 aliquots of 3 ml using cryovials and stored at – 80 °C at the EPHI National reference laboratory for Clinical Chemistry until the analysis is done.

Biochemical analysis (glucose, cholesterol, triglycerides, high-density lipoprotein (HDL-cholesterol and low-density lipoprotein (LDL-cholesterol) were analyzed using Cobas 6000^®^ (Roche Diagnostics GmbH, Mannheim, Germany). Two-level of quality control (PreciControl Clini Chem Multi 1 and 2) were analyzed during the biochemical analysis series. In addition, the reference lab for Clinical Chemistry is an accredited laboratory by Ethiopia National Accreditation Office (ENAO). The laboratory analyses were done by well trained and experienced professionals with strictly followed laboratory standard operating procedures.

### Criteria for metabolic syndrome classification

The definition criteria were based on the International Diabetes Federation (IDF)^14^ and the National Cholesterol Education Program Adult Treatment Panel (NCEP ATP III)^15^ (Table 1). Central obesity can also be measured with WHtR which is more convenient to use for all individuals with variable ethnic groups in different populations and both sexes having a single threshold value of 0.5.

**Table 1:** Classification criteria for metabolic syndrome based on IDF and NCEP ATP III.

Components	IDF criteria	NCEP ATP III criteria
Components	Central obesity Plus at least any two of the other abnormalities	Any two or more of the following abnormalities
Abdominal obesity	Waist circumference ≥ 90 cm for men ≥ 80 cm for women	Waist circumference ≥ 102 cm for men ≥ 88 cm for women
Dyslipidaemia	Triglycerides ≥ 150 mg/dl Low HDL ≤ 40 mg/dl for men and ≤ 50 mg/dl for women	Triglycerides ≥ 150 mg/dl Low HDL ≤ 40 mg/dl for men and ≤ 50 mg/dl for women
Blood pressure	Systolic blood pressure ≥ 130 mmHg and/or diastolic blood pressure ≥ 85 mmHg or current use of antihypertensive drugs)	Systolic blood pressure ≥ 130 mmHg and/or diastolic blood pressure ≥ 85 mmHg or current use of antihypertensive drugs)
Fasting Blood Glucose	Fasting plasma glucose ≥ 100 mg/dl	Fasting plasma glucose ≥ 100 mg/dl

### Data processing and analysis

Descriptive data analyses were performed, along with bivariate and multivariate logistic regression for sex, age, smoking status, alcohol drinking status, physical activity level, khat chewing Status, fruit and vegetable consumption, BMI, WHtR, raised blood pressure, lipid profile (normal vs abnormal) as a confounding factor. All factors with a p value < 0.2 in the bivariate analysis were further analyzed with multivariate logistic regression analysis. The data were analyzed using SPSS software version 20.00 (SPSS Inc. Chicago, IL, USA), and p values < 0.05 were considered statistically significant.

### Ethical approval

Ethical clearance was obtained from Addis Ababa University Biochemistry Department ethics and research committee (DRERC). All study participants provided written informed consent. The identity of participants was not revealed, and an identification number was allocated. All methods used were also performed by the relevant guidelines and regulations.

## Results

### General characteristics of the study participants

A total of 450 (232 males, 218 females) study participants were included from all staff members of EPHI. In this study with 46% and 36% of males and females, the study participants were between 29 and 39 years, respectively. Half of the study participants were married and completed college/University completed and around 24% of the study participants during the study period had less than 1500 birr income per month (Table 2).

**Table 2:** Socio-demographic characteristics of the study participants stratified by sex, EPHI, Addis Ababa, Ethiopia, 2018 (n = 450).

Characteristics	Total	Sex		p value
		Male n (%)	Female n (%)	
Study participant, n (%)	450	232 (51.6)	218 (48.4)	
Mean age, y (SD)	36.5 (10)	38 (10)	35 (10)	0.018
**Age of respondent, n (%)**				
18–28	97 (21.6)	34 (14.7)	63 (28.9)	0.006
29–38	186 (41.3)	107 (46.1)	79 (36.2)	
39–48	96 (21.3)	52 (22.4)	44 (20.2)	
49–58	55 (12.2)	29 (12.5)	26 (11.9)	
59–69	16 (3.6)	10 (4.3)	6 (2.8)	
**Marital status, n (%)**				
Never married	156 (34.7)	73 (31.5)	83 (38.1)	< 0.005
Married	265 (58.9)	156 (67.2)	109 (50.0)	
Separated/divorced/widowed	29 (6.4)	3 (1.3)	26 (11.9)	
**Level of educational status, n (%)**				
Less than primary school	31 (6.9)	9 (3.9)	22 (10.1)	< 0.005
Primary school completed	48 (10.7)	21 (9.1)	27 (12.4)	
Secondary school completed	65 (14.4)	24 (10.3)	41 (18.8)	
College/university completed	220 (48.9)	114 (49.1)	106 (48.6)	
Post graduate degree	86 (19.1)	64 (29.6)	22 (10.1)	
**Quartile of income per month, n (%)**				
Quartile1	107 (23.8)	27 (11.6)	80 (36.7)	< 0.005
Quartile2	117 (26)	50 (21.6)	67 (30.7)	
Quartile3	108 (24)	72 (31.0)	36 (16.5)	
Quartile4	118 (26.2)	83 (35.8)	35 (16.1)	

*Quartile 1* =  < *1500 Birr, Quartile 2* = *1500–3173-birr, Quartile 3* = *3174–6676-birr Quartile 4* =  > *6677 birr.*

### Distribution of behavioural, clinical and biochemical characteristics

About two-thirds of the study participants declared a moderate level of physical activity, but about 4% were smokers, 67% consumed alcohol, 4% chew Khat, and less than 1% consumed fruits and vegetables according to WHO criteria. In this study, 2% had hyperglycemia (≥ 126 mg/dl), 24% had high blood pressure, 19% had high serum triglycerides level (≥ 150 mg/dl), and 25% had a high LDL cholesterol level (≥ 130 mg/dl). Significant differences were observed between male and female and smoking status, alcohol intake status, khat chewing status, BMI, blood pressure, CO-based on IDF, and CO based on NCEP-ATPII (p < 0.05) (Table 3).

**Table 3:** Prevalence of behavioural clinical and biological characteristics of the study participant, EPHI, Addis Ababa, Ethiopia, 2018 (n = 450).

Characteristics	N (%) of total	Sex		p value
		Male n (%)	Female n (%)	
**Smoking status, n (%)**				
Never smoke	405 (90)	190 (81.9)	215 (98.6)	< 0.005
Current smoker	19 (4.2)	18 (7.8)	1 (0.5)	
Previous smoker	26 (5.8)	24 (10.3)	2 (0.9)	
**Alcohol intake status, n (%)**				
No	150 (33.3)	60 (25.9)	90 (41.3)	0.001
Yes	300 (66.7)	172 (74.1)	128 (58.7)	
**Physical activity level, n (%)**				
Vigorous	133 (29.6)	74 (31.9)	59 (27.1)	0.365
Moderate	295 (65.6)	149 (64.2)	146 (67.0)	
Low	22 (4.8)	9 (3.9)	13 (4.9)	
**Khat chewing status, n (%)**				
Never chewed	379 (84.2)	166 (71.6)	213 (97.7)	< 0.005
Current chewer	19 (4.2)	19 (8.2)	0 (0)	
Previous chewer	52 (11.6)	47 (20.3)	5 (2.3)	
**Serving of fruit and vegetable per day (WHO) recommendation**				
≥ 5	2 (0.4)	1 (0.4)	1 (0.5)	0.965
< 5	448 (99.6)	231 (99.6)	217 (99.6)	
**BMI (kg/m**^**2**^**)**				
Normal	252 (56)	128 (55.2)	124 (56.9)	0.001
Overweight	167 (37.1)	97 (41.8)	70 (32.1)	
Obese	31 (6.9)	7 (3.0)	24 (11.0)	
**Blood pressure, mmHg**				
Normal	344 (76.4)	172 (74.1)	172 (78.9)	0.019
Raised blood pressure	106 (23.6)	60 (25.9)	46 (21.1)	
**Lipid profiles**				
Cholesterol < 200 mg/dl	323 (71.8)	158 (68.1)	165 (75.7)	0.074
Cholesterol ≥ 200 mg/dl	127 (28.2)	74 (31.9)	53 (24.3)	
Triglyceride < 150 mg/dl	363 (80.7)	161 (64.4)	202 (92.7)	< 0.005
Triglyceride ≥ 150 mg/dl	87 (19.3)	71 (30.6)	16 (7.3)	
Normal HDL mg/dl	264 (58.7)	141 (60.8)	123 (56.4)	0.349
Low HDL mg/dl	186 (41.3)	91 (39.2)	95 (43.6)	
Normal LDL(< 130) mg/dl	337 (74.9)	166 (71.6)	171 (78.4)	0.092
High LDL (> 130) mg/dl	113 (25.1)	66 (28.4)	47 (21.6)	
**Blood glucose**				
Normal	439 (97.6)	224 (96.6)	215 (98.6)	0.121
Hyperglycemia	11 (2.4)	8 (3.4)	3 (1.4)	
**Dyslipidemia based on NCEP-ATPII**				
Normal	227 (50.4)	113 (48.7)	114 (52.3)	0.447
Dyslipidemia	223 (49.6)	119 (51.3)	104 (47.7.6)	
**CO-based on IDF**				
Normal	89 (19.8)	28 (12.1)	61 (28.0)	< 0.005
Obese	361 (80.2)	204 (87.9)	157 (72.0)	
**CO-based on NCEP-ATPII**				
Normal	307 (68.2)	197 (84.9)	110 (50.5)	< 0.005
Obese	143 (31.8)	35 (15.1)	108 (49.5)	
**CO-based on WHtR**				
Normal	130 (28.9)	68 (29.3)	62 (28.4)	0.839
Obese	320 (71.1)	164 (70.7)	156 (71.6)	

*BMI body mass index, raised blood pressure (SBP ≥ 140 and/or DBP ≥ 90 mmHg and/or on medication), hyperglycemia (fasting blood glucose ≥ 126 mg/dl and/or on medication), *LDL* low-density lipoprotein, *HDL* high-density lipoprotein, HDL low < 40/50 mg/dl (M/F) CO (central obesity waist circumference IDF ≥ 90/80 cm, NCEP-ATP III ≥ 102/88 cm male/female); WHtR (waist to height ratio), CO ≥ 0.5.

### Prevalence of determinant factors for metabolic syndrome

From the study participants, the prevalence of elevated blood pressure, raised fasting blood glucose, dyslipidemia, and central obesity were more prevalent among males and increases with increasing age. Among those consuming alcohol had high blood pressure (27.7% vs 15.3%) and were centrally obese by IDF (84.3% vs 72%) compared to those not consume alcohol From study participants who had high BMI and had WHtR above the cut-off value, elevated blood pressure was prevalent 18/21 (58.1%) and 92/320 (28.8%), respectively. The study also showed that Dyslipidemia was prevalent among overweight 102/167 (61.1%), obese 17/31 (54.8%) and those who had raised hsCRP 174/324 (53.4%). From those participants who had central obesity (80.2%) based on IDF criteria, all participants with 59–69 years age group had central obesity (Table 4).

**Table 4:** Prevalence of blood pressure, blood glucose, lipid profiles abnormalities and central obesity of study participants at EPHI, Addis Ababa, Ethiopia, 2018 (n = 450).

Characteristics	High blood pressure, n (%)	Elevated fasting blood glucose, n (%)	Dyslipidemia, n (%)	Centrally obese by IDF, n (%)
**Sex**				
Male	60 (25.9)	8 (3.4)	119 (51.3)	204 (87.9)
Female	46 (21.1)	3 (1.4)	104 (47.7)	157 (72)
Total	106 (23.6)	11 (2.4)	223 (49.6)	361 (80.2)
**Age**				
18–28	9 (9.3)	0 (0)	46 (47.4)	49 (50.5)
29–38	24 (12.9)	1 (0.5)	88 (47.3)	157 (84.4)
39–48	39 (40.6)	3 (3.1)	58 (60.4)	89 (92.7)
49–58	24 (43.6)	5 (9.1)	26 (47.3)	50 (91.9)
59–69	10 (62.5)	2 (12.5)	5 (32.2)	16 (100)
**Smoking status**				
Never smoked	91 (22.5)	7 (1.7)	202 (49.9)	319 (78.8)
Current smoker	5 (26.3)	3 (15.8)	6 (31.6)	18 (94.7)
Previous smoker	10 (38.5)	1 (3.8)	15 (57.7)	24 (92.3)
**Alcohol consumption**				
No	23 (15.3)	1 (0.7)	74 (49.3)	108 (72)
Yes	83 (27.7)	10 (3.3)	149 (49.7)	253 (84.3)
**Physical activity level**				
Vigorous	36 (27.1)	2 (1.5)	65 (48.9)	114 (83.7)
Moderate	63 (21.4)	9 (3.1)	146 (49.5)	233 (79)
Low	7 (31.8)	0 (0)	12 (54.5)	14 (63.6)
**Khat chewing status of respondent**				
Never chewer	90 (23.7)	8 (2.1)	183 (48.3)	300 (79.2)
Current chewer	2 (10.5)	1 (5.3)	10 (52.6)	16 (84.2)
Previous chewer	14 (26.9)	2 (3.8)	30 (57.7)	45 (86.5)
**Days of fruit and vegetable intake per week**				
≥ 5	7 (19.4)	1 (2.8)	19 (52.8)	28 (77.8)
3–5	19 (30.2)	2 (3.2)	31 (49.2)	46 (73)
< 3	80 (22.8)	8 (2.3)	173 (49.3)	287 (81.8)
**BMI**				
Normal	46 (18.3)	4 (1.6)	104 (41.3)	173 (68.7)
Overweight	42 (25.1)	4 (2.4)	102 (61.1)	158 (94.6)
Obese	18 (58.1)	3 (9.7)	17 (54.8)	30 (96.8)
**WHtR**				
Normal	14 (10.8)	1 (0.8)	45 (34.6)	46 (35.4)
Obese	92 (28.8)	10 (3.1)	178 (55.6)	315 (98.4)
**Raised blood pressure**				
Normal	NA	4 (1.2)	171 (49.7)	198 (73.9)
Hypertensive	NA	7 (6.6)	52 (49.1)	163 (89.6)
**Raised blood glucose**				
Normal	99 (22.6)	NA	219 (49.9)	321 (78.9)
Hyperglycemia	7 (63.6)	NA	4 (36.4)	40 (93)
**Lipid profile**				
Normal	54 (23.8)	7 (3.1)	NA	171 (75.30
Dyslipidemia	52 (23.3)	4 (1.8)	NA	190 (85.2)
**Central obesity by IDF**				
Normal	19 (21.3)	1 (1.1	33 (37.1)	NA
Obese	163 (45.2)	10 (2.8)	190 (52.6)	NA

*HTN* hypertension, raised blood pressure (systolic BP ≥ 130 mmHg, diastolic BP ≥ 85 mmHg), *DM* diabetes mellitus (raised fasting glucose ≥ 100 mg/dl), central obesity (defined by a waist circumference ≥ 94 cm for men and ≥ 80 cm for women), Dyslipidemia (defined by raised triglyceride level ≥ 150 mg/dl and reduced HDL < 40 mg/dl in men and < 50 mg/dl in women). WHtR waist to height ratio (0.5 = normal and ≥ 0.5 obese), *BMI* body mass index.

### Associated factors with raised blood pressure, raised blood glucose, dyslipidemia and central obesity

The results of the logistic regression analyses for raised blood pressure, raised blood glucose, dyslipidemia, and central obesity based on IDF are presented in Table 5. In logistic regression analysis, being female [ADJUSTED OR (AOR) = 0.07; 95% CI 0.02, 0.2], increasing age, overweight, were independent risk factors for central obesity based on IDF. Being a current smoker (AOR = 10.34; 95% CI 2.2, 48.7) and hypertensive (AOR = 4.8; 95% CI 1.24, 18.62) were also independent risk factors for raised fasting blood glucose based on WHO criteria. Overweight and raised WHtR had a significant association with dyslipidemia in a multi logistic regression model with OR as follows overweight [AOR=1.68, (95% CI 1.07–2.63)] and raised WHtR [AOR=1.74, (95% CI 1.07–2.81)].

**Table 5:** Bivariate and multivariate analyses of demographic and clinical risk factors for raised blood pressure, raised blood sugar, dyslipidemia and central obesity of study subjects. EPHI, Ethiopia, Addis Ababa, 2018 (n = 450).

Characteristics	Raised blood pressure		Raised blood glucose		Dyslipidemia		Central obesity based on IDF	
	COR^1^ (95% CI)	AOR^2^ (95% CI)	COR^1^ (95% CI)	AOR^2^ (95% CI)	COR^1^ (95% CI)	AOR^2^ (95% CI)	COR^1^ (95% CI)	AOR^2^ (95% CI)
**Sex**								
Male	1.00	–	1.00	–	1.00	–	1.00	1.00
Female	0.77 (0.5–1.20)	–	0.39 (0.10–1.49)	–	0.87 (0.6–1.25)	–	0.35 (0.22–0.58)	0.07 (0.02–0.2)*
**Age**								
18–28	1.00	1.00		–	1.00	–	1.00	1.00
29–38	1.45 (0.65–3.25)	1.11 (0.48–2.58)		–	1.0 (0.61–1.63)	–	5.30 (3.02–9.30)	2.6 (1.05–6.55)
39–48	6.69 (3.01–14.85)	4.27 (1.8–10.13)*		–	1.69 (0.96–3.0)	–	12.45 (5.24–29.62)	2.78 (0.68–11.5)
49–58	7.57 (3.18–18.04)	4.53 (1.77–11.6)*		–	1.0 (0.51–1.93)	–	9.8 (3.6–26.7)	1.17 (0.25–5.46)
59–69	16.3 (4.8–55.34)	8.62 (2.36–31.5)*		–	0.50 (0.16–1.56)	–	–	–
**Smocking status**								
Never smoked	1.00	–	1.00	1.00	1.00	–	1.00	–
Current smoker	1.23 (0.43–3.51)	–	10.66 (2.51–45)	10.34 (2.2–48.7)*	0.46 (0.17–1.24)	–	4.85 (0.64–36.9)	–
Previous smoker	2.16 (0.95–4.92)	–	2.27 (0.27–19.2)	1.74 (0.2–15.42)	1.37 (0.62–3.06)	–	3.23 (0.75–13.96)	–
**Alcohol drinking status**								
No	1.00	1.00	1.00	–	1.00	–	1.00	–
Yes	0.47 (0.28–0.79)	1.68 (0.96–2.93)	5.14 (0.65–40.5)	–	1.01 (0.68–1.5)	–	2.09 (1.30–3.36)	–
**Physical activity level (WHO recommendation)**								
Vigorous	1.00	–	1.00	–	1.00	–	1.00	1.00
Moderate	0.73 (0.46–1.17)	–	2.06 (0.44–9.67)	–	1.03 (0.68–1.54)	–	0.63 (0.36–1.1)	0.68 (0.35–1.31)
Low	1.23 (0.47–3.33)	–	0.00 (0.00)	–	1.25 (0.51–3.11)	–	0.29 (0.11–0.79)	0.21 (0.06–0.77)
**Khat chewing status of respondent**								
Never chewer	1.00	–	1.00	–	1.00	–	1.00	–
Current chewer	0.38 (0.09–1.67)	–	2.58 (0.31–21.7)	–	1.19 (0.47–2.99)	–	1.4 (0.4–4.94)	–
Previous chewer	1.18 (0.61–2.28)	–	1.85 (0.38–8.98)	–	1.46 (0.81–2.62)	–	1.69 (0.73–3.89)	–
**Days of fruit and vegetable intake per week (WHO recommendation)**								
≥ 5	1.00	–	1.00	–	1.00	–	1.00	–
3–5	1.8 (0.67–4.79)	–	1.15 (0.1–13.11)	–	0.87 (0.38–1.97)	–	0.77 (0.29–2.02)	–
< 3	1.22 (0.52–2.9)	–	0.82 (0.1–6.72)	–	0.87 (0.44–1.73)	–	1.28 (0.56–2.94)	–
**Body mass index (BMI)**								
Normal	1.00	1.00	1.00	1.00	1.00	1.00	1.00	1.00
Overweight	1.51 (0.94–2.42)	0.86 (0.50–1.50)	1.52 (0.37–6.17)	1.31 (0.31–5.52)	2.23 (1.5–3.33)	1.68 (1.07–2.63)*	8.01 (3.89–16.5)	4.87 (2.23–10.6)*
Obese	6.2 (2.84–13.55)	2.31 (0.95–5.59)	6.64 (1.41–31.2)	3.18 (0.57–17.8)	1.73 (0.82–3.66)	1.25 (0.57–2.74)	13.4 (1.84–102)	9.29 (1.07–80.71)*
**WHtR**								
Normal (< 0.5)	1.00	1.00	1.00	–	1.00	1.00	1.00	1.00
Obese (≥ 0.5)	3.34 (1.83–6.12)	1.66 (0.81–3.40)	4.16 (0.53–32.8)	–	2.37 (1.55–3.62)	1.74 (1.07–2.81)*	115 (44–298)	399 (82–1080)*
**Raised blood pressure (≥ 140/90 mmHg)**								
Normal	NA	NA	1.00	1.00	1.00	–	1.00	1.00
Hypertension	NA	NA	6.10 (1.72–20.9)	4.8 (1.24–18.62)*	0.97 (0.63–1.51)	–	4.43 (1.98–9.91)	1.94 (0.57–6.55)
**Raised blood glucose level (≥ 126 mg/dl)**								
Normal	1.00	1.00	NA	NA	1.00	–	1.00	–
Hyperglycemia	6.01 (1.72–20.95)	2.20 (0.56–8.61)	NA	NA	0.57 (0.17–1.99)	–	2.51 (0.32–19.85)	–
**Lipid profile**								
Normal	1.00	–	1.00	–	NA	NA	1.00	1.00
Dyslipidemia	0.97 (0.63–1.51)	–	0.57 (0.17–1.99)	–	NA	NA	1.89 (1.17–3.04)	− 1.04 (0.45–2.4)

Raised blood pressure (systolic BP ≥ 130 mmHg, diastolic BP ≥ 85 mmHg) DM: diabetes mellitus (raised fasting glucose ≥ 100 mg/dl), central obesity (defined by a waist circumference ≥ 94 cm for men and ≥ 80 cm for women), Dyslipidemia (defined by raised triglyceride level ≥ 150 mg/dl and reduced HDL < 40 mg/dl in men and < 50 mg/dl in women).WHtR (waist to height ratio, COR (crude odds ratio), AOR (adjusted odds ratio).

*Significant during multi logistic analysis.

### Proportions of metabolic syndrome

**Figure 1:**
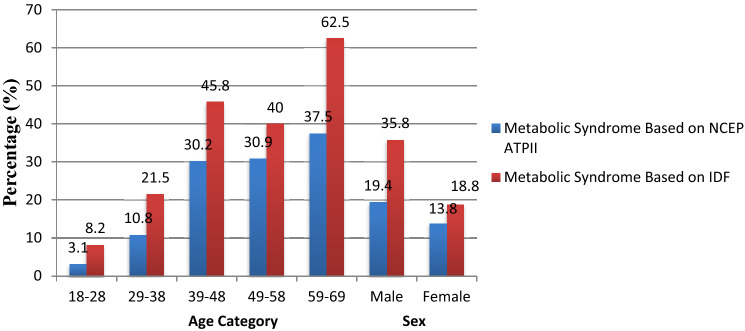
Prevalence of Metabolic Syndrome Based on NCEP ATP III and IDF criteria stratified by Age Group and Sex of Participants EPHI, Addis Ababa, Ethiopia, 2018.

In this study, 27.6% and 16.7% of the study participants had MetS based on IDF and NCEP ATP III, respectively. The prevalence of MetS based on both IDF and NCEP criteria was higher in males, 83/232 (35.8%) and 45/232 (19.4%), respectively compared to females. The prevalence of MetS increases with age increases and the age group between 59 and 69 years of age had the highest prevalence based on both criteria (Fig. 1).

### Risk factors of metabolic syndrome

In multivariate analysis, sex, age, BMI, raised blood glucose; raised blood pressure and dyslipidemia were shown to be significant risk factors for metabolic syndrome based on IDF criteria (Table 5). The odd of developing metabolic syndrome is 32% smaller for females [AOR= 0.32, (95% CI 0.16–0.64)]. Age groups 39–48, 49–58, 59–69 and overweight were individual predictors for metabolic syndrome [AOR = 5.38, (1.72–16.8)], [AOR = 4.0, (1.09–14.7)], [AOR = 81.2 (9.4–669)] and [AOR = 4.67, (95% CI 2.27–17.6)], respectively. From our research result raised blood pressure and blood glucose and dyslipidemia were also significant risk factors for metabolic syndrome with AOR = 28 (95% CI 9.46–86.9), AOR = 126 (95% CI 6.7–2374) and AOR = 210 (95% CI 52–849), respectively. Almost similar results were observed using ATP III criteria on the significance of risk factors for metabolic syndrome. Central obesity was the constant component for IDF criteria while according to ATP III criteria it was a statistically significant risk factor for metabolic syndrome with [AOR = 9.56 (95% CI 4.11–22.3)]. Regarding sex difference, there was no statistical association for metabolic syndrome based on ATP III criteria (Table 6).

**Table 6:** Bivariate and multivariate analyses of risk factor components for metabolic syndrome of study subjects. EPHI, Addis Ababa, Ethiopia, 2018 (n = 450).

Characteristics	MS-IDF		MS-NCEP ATP IIII	
	COR (95% CI)	AOR (95% CI)	COR (95% CI)	AOR (95% CI)
**Sex**				
Male	1.00	1.00	1.00	–
Female	0.42 (0.27–0.64)	0.32 (0.16–0.64)*	0.66 (0.4–1.09)	–
**Age of respondents**				
18–28	1.00	1.00	1.00	1.00
29–38	3.05 (1.36–6.81)	2.1 (0.74–5.95)	3.78 (1.09–13.0)	1.24 (0.48–7.6)
39–48	9.41 (4.12–21.5)	5.38 (1.72–16.8)*	13.6 (3.97–46.4)	3.84 (0.79–18.8)
49–58	7.42 (3.0–18.29)	4.0 (1.09–14.7)*	14 (3.88–50.4)	3.3 (0.6–18.05)
59–69	18.5 (5.34–64.3)	81.2 (9.4–669)*	18.8 (4.06–86.9)	15 (1.74–129)*
**Smocking status**				
Never smoke	1.00	1.00	1.00	–
Current smoker	1.69 (0.65–4.4)	1.46 (0.15–14)	2.55 (0.94–6.97)	–
Previous smoker	2.89 (1.3–6.44)	0.88 (0.23–3.38)	2.04 (0.82–5.05)	–
**Alcohol drinking status of respondent**				
No	1.00	1.00	1.00	–
Yes	1.72 (1.08–2.74)	1.28 (0.62–2.63)	1.72 (0.97–3.04)	–
**Physical activity level (WHO recommendation)**				
Vigorous	1.00	–	1.00	–
Moderate	0.93 (0.59–1.47)	–	0.84 (0.49–1.44)	–
Low	0.94 (0.34–2.58)	–	1.34 (0.45–3.98)	–
**Khat chewing status of respondent**				
Never Chewed	1.00	–	1.00	1.00
Current Chewer	1.32 (0.49–3.58)	–	1.51 (0.48–4.7)	1.41 (0.21–9.3)
Previous Chewer	1.79 (0.98–3.28)	–	2.08 (1.06–4.08)	1.66 (0.66–4.58)
**Serving of fruit and vegetable per day (WHO) recommendation**				
≥ 5	1.00	–	–	–
< 5	0.38 (0.02–6.1)	–	–	–
**Body mass index (BMI)**				
Normal	1.00	1.00	1.00	1.00
Overweight	4.0 (2.77–7.0)	4.67 (2.27–9.6)*	9.04 (4.65–17.6)	5.28 (2.12–13.7)*
Obese	5.45 (2.48–11.9)	2.2 (0.6–8.05)	11 (4.3–28.1)	1.75 (0.49–6.21)
**Blood pressure**				
Normal	1.00	1.00	1.00	1.00
Hypertensive	6.05 (3.77–9.7)	28 (9.46–86.9)*	3.75 (2.22–6.32)	4.55 (1.88–11.01)*
**Blood glucose**				
Normal	1.00	1.00	1.00	1.00
Hyperglycemia	28.5 (3.61–225)	126 (6.7–2374)*	9.55 (2.72–33.5)	41.5 (4.75–362.9)*
**Dyslipidemia based on NCEP-ATPII**				
Normal	1.00	1.00	1.00	1.00
Dyslipidemia	14.8 (8.12–27)	210 (52–849)*	13.9 (6.2–30.8)	85.5 (21.9–332)*
**Waist circumference**				
Normal	NA	NA	1.00	1.00
Centrally obese	NA	NA	7.05 (4.09–12.16	9.56 (4.11–22.3)*

*MS-IDF* metabolic syndrome based on IDF (waist circumference  ≥  94/80 cm plus any two of the following (1) raised blood pressure ≥ 130/85, (2) raised fasting blood glucose ≥ 100 mg/dl, (3) fasting triglyceride ≥ 150 mg/dl and (4) HDL < 40/50 for male/female. MS-NCEP ATPII metabolic syndrome based on ATP III defined by any three of the following (1) waist circumference ≥ 102/88 male/female; (2) hypertriglyceridemia: serum TG ≥ 150 mg/dl, (3) low HDL-C < 40/50 mg/dl male/female, (4) hypertension: SBP ≥ 130 mmHg or DBP ≥ 85 mmHg and (5) fasting plasma glucose ≥ 110 mg/dl.

*Significant during multi logistic analysis.

### Summary of combined cardiometabolic risk factors

**Figure 2:**
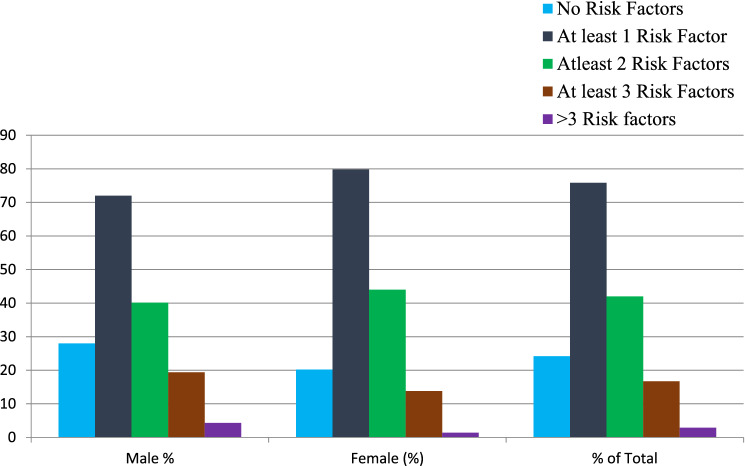
Summary of combined risk factors by sex of study participant based on ATP III definition, EPHI, Addis Ababa, Ethiopia, 2018 (n = 450).

According to NCEP ATP III criteria, among the total study participants, only 109/450 (24.2%) had no risk factors for cardiometabolic diseases; out of which 65/232 (28%) were males. As shown in Fig. 2, more than three risk factors were more prevalent among male participants 10/232 (4.3%) as compared to female 3/218 (1.4%), having a crude percentage of 13/450 (2.9%). Nearly one-fourth of the study participants, 341/450 (75.8%) had at least one risk factor for metabolic syndrome and cardiometabolic risks. About 189/450 (42%) of the study participants had at least two or more risk factors for metabolic syndrome; of which the prevalence is higher among females 96/218 (44%) as compared to males 93/232 (40.1%). The prevalence of having at least three risk factors for metabolic syndrome was higher among males 45/232 (19.4%) from the total prevalence of 75/450 (16.7%) (Fig. 2).

## Discussion

MetS is a constellation of different risk factors associated with a 5-fold increase in the incidence of Type 2 diabetes and a 2–3-fold increase in the incidence of CVDs^16^. Based on our findings the prevalence of overweight or obese, of were found higher than other studies conducted in Ethiopia national survey (1.2% obese and 5.2% overweight)^17^, northern Ethiopia, Mekele (4.1% obese and 26% overweight)^18^ and Northwest Ethiopia, Jimma obese (5.1%) and overweight (10.4%)^19^.

Hypertension, the third most prevalent component (23.6%) for metabolic syndrome was higher than those reported in earlier studies 15.8% conducted in Ethiopia in 2015 national survey^17^, 9.3% at Gilgel Gibe field research center^20^ and 20% in male and 14% in female among working adults in Addis Ababa^21^. Possible explanations for the difference are stress conditions, lifestyle and genetic differences, and alcohol consumption status might have contributing factors. Our study participants had a high prevalence in alcohol consumption status as compared to other studies. The association of alcohol consumption with an increased incidence of hypertension was explained by different studies^22,23^. This showed that appropriate interventions were needed to reduce the burden of alcohol use, which could help to lower blood pressure levels^24^. The prevalence of hypertension was also higher when compared to a study done in Angola, 17.9%^25^ but lower in comparisons with studies done in Eastern Ethiopia 28.3%^26^, Nigeria Lagos 38.2%^27^ and in Ghana 55.3%^28^. The possible explanation for the disparities was due to family history, socio-demography, attitude, and awareness and geographic location and/or maybe lifestyle of study participants.

The result of our research showed that the prevalence of diabetes mellitus was 2.4%, which is in line with a study done in rural Koladiba town of northwest Ethiopia^29^. Our result was slightly similar to the 2010 global estimate of the prevalence of diabetes in the Ethiopian population, 2.0%^30^ and a study done in the South Western Nigeria population 2.5%^31^. However, our result was found lower than other studies like Ethiopian national crude prevalence rate 3.2%^32^ and study done in Northern Ethiopia 10.1%^32^. This may be due to biochemical tests used to define the prevalence of diabetes. In our study, we had used only fasting blood glucose but the study done by Gebremariam et al.^18^ used a combination of FBG and HgA1c, which results in observed prevalence differences.

Dyslipidemia, especially low HDL levels with 41.3% was the second most prevalent finding in our study participants. The prevalence of low HDL in our study is in line with other studies^31,33,34^. On the contrary, a higher prevalence of low HDL was observed in the Ethiopian national survey (68%) and finding among public employees in northern Ethiopia (71.3%)^18,32^. Environmental factors, physical activity status, nutrient intake and sample size and age of study participants may be used as part of an explanation for this difference.

Regarding the prevalence of hypertriglyceridemia, that is (19.3%) in our study is nearly similar to a result reported in the Ethiopian national survey. But higher prevalence is found in different studies^18,34,35^. Dietary intake, level of physical activity, lifestyle difference, and level of awareness may be part of a possible explanation for this variation.

Abdominal obesity drives the development of cardiometabolic risks through altered secretion of adipocyte-derived active substances called adipokines, including free fatty acids, adiponectin, interleukin-6, tumour necrosis factor-alpha, and plasminogen activator inhibitor-1, and through exacerbation of insulin resistance and associated cardiometabolic risk factors^36^. In the present study, we have found that elevation of waist circumference based on IDF criteria was the most prevalent (80.2%) that was the superior component to yield a larger magnitude for metabolic syndrome. This result is higher than the community-based study done among Andean highlanders (75.9%)^37^ and the study done in South African Asian Indians who found a prevalence of (73.1%) even though harmonized criteria were used^38^. This may be due to differences in sample size, level of physical activity difference, and dietary intake. Concerning sex-difference, it is noted that males had a higher frequency of central obesity (87.9%). The reason for this difference may be the majority (65%) of female participants were younger as compared to males (35%) and central obesity increases with increasing age^39^.

Findings from this study showed that the prevalence of metabolic syndrome among staff members of EPHI was 16.7% using NCEP ATP III criteria while the IDF criteria yielded a higher prevalence of 27.6%. This higher prevalence of metabolic syndrome based on IDF criteria was due to a higher prevalence of central obesity which is one of the pre-request criteria for defining metabolic syndrome. Based on IDF criteria our result was fairly comparable to studies conducted in different regions and countries^40–44^. The prevalence of metabolic syndrome in our study was less than from other studies^35,45–47^. Differences in the age of study subjects, sample size, socioeconomic status, residence & lifestyle, dietary intake, and physical activity may contribute to the different prevalence of metabolic syndrome in these different studies.

The high prevalence of metabolic syndrome has been linked to urbanization, westernization, nutritional and epidemiological transition^48^. Our result was also showed lower prevalence than the recent study conducted in Northern Ethiopia involving public employees in Mekele, which found a prevalence of metabolic syndrome was 40% using IDF criteria^18^. The explanation for this discordant may be due to the environmental and sampling methods in which we had used random sampling. However, the finding in this study was higher than other community-based studies using both NCEP ATP III and IDF criteria^17,19,21^. Our result had found comparably higher prevalence than studies conducted among adults in the rural area of West China (10.8%) and health professionals in Brazil (4.5%)^49,50^. This could be due to differences in socioeconomic backgrounds, lifestyle variations and ethnic differences.

The result also showed that the prevalence of metabolic syndrome, based on IDF criteria was 35.8% in males and 18.8% in females. This was in line with the study reported in Colombia who observe that the prevalence of metabolic syndrome in males was three times higher than in females^33^. The possible explanation for the higher prevalence of metabolic syndrome in males is due to the majority of female participants are younger as compared to males^39^. We have also found older age was significantly associated with metabolic syndrome. The other possible explanation for higher metabolic syndrome prevalence in males can be because of central obesity which was the primarily prevalent component in males (72%) than females (28%). However, in contradiction with our result, other studies have reported a higher prevalence of metabolic syndrome among females. The prevalence of metabolic syndrome was also found significantly higher in older age, which is in line with other studies^19,51^. The reason is that ageing is characterized by a progressive deterioration in physiological functions and metabolic processes that generate reactive oxygen species as a by-product of biological oxidation. The oxidative damage of reactive oxygen species induces cellular dysfunction, which plays an important role in many pathological conditions like chronic low-level inflammation-induced metabolic syndrome^52^. The predisposing factors to develop metabolic syndrome, includes being overweight [OR 4.67, (95% CI 2.27–9.6)], having raised blood pressure [OR 28, (95% CI 9.46–86.9)], raised fasting blood glucose [OR 126, (95% CI 6.7–2374)] and dyslipidemia [OR 210, (95% CI 52–849)]. These were also in line with other studies^19,33,51^. Overweight characterized by unbalanced energy intake and expenditure could result in continued elevation of blood glucose level^53,54^. Thus, it further results in hyper-secretion of insulin and leading to insulin resistance over time. Once insulin resistance occurs in different target organs for metabolic process dysregulation could be initiated such as lipid profile abnormalities, endothelial dysfunction, and inflammatory reactions^55,56^.

This result revealed that smoking habits, alcohol consumption, physical activity status and serving of fruit and vegetables per day were not individual predictors for metabolic syndrome. These findings were consistent with other researcher’s reports^57,58^. Another finding was in contrary found that smoking, alcohol use, fruit, and vegetable consumption were statistically significant factors for metabolic syndrome^48^. The discordant between smoking and alcohol use with these research findings might be due to the amount and type of alcohol and smoking products taken by the study populations might be different. However, sex, age, BMI, raised blood pressure, raised blood glucose, dyslipidemia and raised hsCRP had statistical significance with metabolic syndrome in bivariate analysis. After adjusting confounders in logistic regression only age, BMI, elevated blood glucose, high blood pressure and dyslipidemia were independent predictors for metabolic syndrome. This was also in line with different studies^50,59^. Three fourth of the participants had at least one component for metabolic syndrome. The prevalence of central obesity expressed as increased waist circumference was the first ranked abnormality, followed by low HDL and raised blood pressure which is acquiesced with many researchers^35,60^. It is assumed that the luxurious lifestyle lies behind abdominal obesity and dyslipidemia for the most prevalent components of metabolic syndrome^61^. High prevalence of abdominal/central obesity, low HDL and raised blood pressure emphasizes the susceptibility of the study population to CVD and Type 2 DM, especially in older age. Controlling body mass and fat with better physical activity and an appropriate diet are important to reduce the risk of CVDs^62^. The prevalence also has been linked to urbanization, westernization, nutritional and epidemiological transition and this calls for urgent action by the policymakers and health managers to further emphasize the need for routine screening for all the components of Metabolic syndrome.

## Conclusion

From this study, it is possible to conclude the following: the prevalence of metabolic syndrome and its components were significantly high among the study population as compared to other studies like the country national survey. Central obesity, followed by dyslipidemia and hypertension were the most frequent components of metabolic syndrome. The prevalence of hypertension was found substantial as compared to the national survey report. Being male, over 39 years old, overweight, raised blood pressure elevated fasting blood glucose and dyslipidemia were significantly associated with metabolic syndrome. Twenty-four percent of the study participants were free from any risk factors for metabolic syndrome. About 16.7% of the study participants had ≥ 3 risk factors based on NCEP ATP III defining criteria.

### Limitation of the study

The study employed a cross-sectional study design which could not conclude causality and effects. Moreover, this finding may not be generalized to a broader Ethiopian population since our study participants were an employee of a specific organization.

## Data Availability

The whole data supporting this study are included within the manuscript.

## References

[1] Ekpenyong T, Udokang CE, Akpan NE, Samson EE (2012). Double burden, non-communicable diseases and risk factors evaluation in Sub-Saharan Africa: The Nigerian experience. Eur. J. Sustain. Dev..

[2] World Health Organization (2017). Non-Communicable Diseases Progress Monitor 2017.

[3] World Health Organization (2014). Global status Report on Noncommunicable Diseases.

[4] Alzeidan R, Rabiee F, Mandil A, Hersi A, Fayed A (2016). Non-communicable disease risk factors among employees and their families of a Saudi University: An epidemiological study. PLoS One.

[5] Kaukua J, Turpeinen A, Uusitupa M, Niskanen L (2001). Clustering of cardiovascular risk factors in type 2 diabetes mellitus: Prognostic significance and tracking. Diabetes Obes. Metab..

[6] Paper, R. The cardiometabolic syndrome and cardiovascular disease (2006). 10.1111/j.0197-3118.2006.05452.x 17675903

[7] Ala, A., Adelin, A. & Michèle, G. Cardiometabolic syndrome.

[8] Carney R, Cotter J, Bradshaw T, Firth J, Yung AR (2016). Cardiometabolic risk factors in young people at ultra-high risk for psychosis: A systematic review and meta-analysis. Schizophr. Res..

[9] Krishnadas R, Jauhar S, Telfer S, Shivashankar S, Mccreadie RG (2012). Nicotine dependence and illness severity in schizophrenia. Brit. J. Psychiatry.

[10] National Institutes of Health (2000). The Practical Guide: Identification, Evaluation, and Treatment of Overweight and Obesity in Adults.

[11] Ashwell M, Gibson S (2009). Waist to height ratio is a simple and effective obesity screening tool for cardiovascular risk factors: Analysis of data from the british national diet and nutrition survey of adults aged 19–64 years. Obes. Facts.

[12] WHO. *Emro, 5–17 Years Old*, pp. 2–3.

[13] Bonita, R., Winkelmann, R., Douglas, K. A. & de Courten, M. The WHO stepwise approach to surveillance (steps) of non-communicable disease risk factors. In *Global Behavioral Risk Factor Surveillance*, 2003.

[14] Zimmet P, Magliano D, Matsuzawa Y, Alberti G, Shaw J (2005). The metabolic syndrome: A global public health problem and a new definition. J. Atheroscler. Thromb..

[15] Third Report of the National Cholesterol Education Program, Third Report of the National Cholesterol Education Program (NCEP) Expert Panel on, 01-3670, 2001. 10.1001/jama.285.19.2486. 10.1001/jama.285.19.2486 11368702

[16] Epidemiology F (2009). Harmonizing the Metabolic Syndrome International Atherosclerosis Society; and International Association for the study of obesity. Circulation.

[17] Ethiopia steps report on risk factors for non-communicable disease and prevalence of selected NCDs Ethiopia steps report on risk factors for chronic non-communicable diseases and, no. December, 2016.

[18] Gebremariam LW, Chi C, Yatsuya H, Haregot E (2018). Non-communicable disease risk factor profile among public employees in a regional city in northern Ethiopia. Sci. Rep..

[19] Rajesh, P. N., Mossie, A. & Mezgebu, Y. Prevalence Of Metabolic Syndrome And Its Components In Jimma, vol. 3, no. 3, pp. 1685–1704, 2016. 10.18535/ijmsci/v3i3.7.

[20] Tefera TB, Woldemichael K, Tessema F, Alemseged F (2015). Epidemiology of non-communicable disease risk factors among adults residing in gilgel gibe field research. Eur. J. Prev. Med..

[21] Tran A (2011). Prevalence of metabolic syndrome among working adults in Ethiopia. Int. J. Hypertens..

[22] Briasoulis A, Agarwal V, Messerli FH (2012). Alcohol consumption and the risk of hypertension in men and women: A systematic review and meta-analysis. J. Clin. Hypertens..

[23] Zhao F, Liu Q, Li Y, Feng X, Chang H, Lyu J (2020). Association between alcohol consumption and hypertension in Chinese adults: Findings from the CHNS. Alcohol.

[24] Rehm J (2017). Towards new recommendations to reduce the burden of alcohol-induced hypertension in the European Union. BMC Med..

[25] Manuel, V., Manuel, A. & Béu, G. Prevalence of cardiovascular risk factors among workers at a private tertiary center in Angola, pp. 497–503, 2016. 10.2147/VHRM.S120735 PMC5167297 28008265

[26] Asresahegn H, Tadesse F, Beyene E (2017). Prevalence and associated factors of hypertension among adults in Ethiopia: A community based cross-sectional study. BMC Res. Notes.

[27] Daniel OJ, Adejumo OA, Adejumo EN, Owolabi RS, Braimoh RW (2013). Prevalence of hypertension among urban slum dwellers in Lagos, Nigeria. J. Urban Health.

[28] June, M.*et al.* Prevalence and awareness of Hypertension among urban and rural Adults in the Keta Municipality, Ghana, vol. 3, no. 3, pp. 155–163, 2017.

[29] Worede A, Alemu S, Gelaw YA, Abebe M (2017). The prevalence of impaired fasting glucose and undiagnosed diabetes mellitus and associated risk factors among adults living in a rural Koladiba town, northwest Ethiopia. BMC Res. Notes.

[30] Shaw JE, Sicree RA, Zimmet PZ (2010). Global estimates of the prevalence of diabetes for 2010 and 2030. Diabetes Res. Clin. Pract..

[31] Oladapo OO, Salako L, Sodiq O, Shoyinka K, Adedapo K, Falase AO (2010). A prevalence of cardiometabolic risk factors among a rural Yoruba south-western Nigerian population: A population-based survey. Cardiovasc. J. Afr..

[32] Y. F. Gebreyes *et al.*, Prevalence of high bloodpressure, hyperglycemia, dyslipidemia, metabolic syndrome and their determinants in Ethiopia: Evidences from the National NCDs STEPS, pp. 1–18, 2018. 10.1371/journal.pone.0194819 PMC5942803 29742131

[33] Martínez-Torres J (2017). A cross-sectional study of the prevalence of metabolic syndrome and associated factors in colombian collegiate students: The fuprecol-adults study. Int. J. Environ. Res. Public Health.

[34] Amin TT, Al Sultan AI, Mostafa OA, Darwish AA, Al-Naboli MR (2014). Profile of non-communicable disease risk factors among employees at a Saudi University. Asian Pac. J. Cancer Prev..

[35] Obeidat AA, Ahmad MN, Haddad FH, Azzeh FS (2015). Alarming high prevalence of metabolic syndrome among Jordanian adults. Pak. J. Med. Sci..

[36] Farzadfar F (2011). National, regional, and global trends in serum total cholesterol since 1980: Systematic analysis of health examination surveys and epidemiological studies with 321 country-years and 3·0 million participants. Lancet.

[37] Herrera-Enriquez K, Narvaez-Guerra O (2017). Discordance of metabolic syndrome and abdominal obesity prevalence according to different criteria in Andean highlanders: A community-based study. Diabetes Metab. Syndr. Clin. Res. Rev..

[38] Prakaschandra R, Naidoo DP (2017). Increased waist circumference is the main driver for the development of the metabolic syndrome in South African Asian Indians. Diabetes Metab. Syndr. Clin. Res. Rev..

[39] Kuk JL, Ardern CI (2010). Age and sex differences in the clustering of metabolic syndrome factors: Association with mortality risk. Diabetes Care.

[40] Aryal N, Wasti SP (2016). The prevalence of metabolic syndrome in South Asia: A systematic review. Int. J. Diabetes Dev. Ctries..

[41] Kingue S, Rakotoarimanana S, Rabearivony N, Bompera FL (2017). Prevalence of selected cardiometabolic risk factors among adults in urban & semi-urban hospitals in four sub-Saharan African countries. Cardiovasc. J. Afr..

[42] Oguoma VM, Nwose EU, Richards RS (2015). Prevalence of cardio-metabolic syndrome in Nigeria: A systematic review. Public Health.

[43] Cameron AJ, Magliano DJ, Zimmet PZ, Welborn T, Shaw JE (2007). The Metabolic Syndrome in Australia: Prevalence using four definitions. Diabetes Res. Clin. Pract..

[44] Vidigal, F. D. C., Bressan, J., Babio, N. & Salas-salvadó, J. Prevalence of metabolic syndrome in Brazilian adults: A systematic review, 2013. 10.1186/1471-2458-13-1198 PMC3878341 24350922

[45] Kaduka LU (2012). Prevalence of metabolic syndrome among an urban population in Kenya. Diabetes Care.

[46] Gundogan K (2013). Metabolic syndrome prevalence according to ATP III and IDF criteria and related factors in Turkish adults. Arch. Med. Sci..

[47] Adeyemi YA, Onabanjo OO, Sanni SA, Ugbaja RN, Afolabi DO, Oladoyinbo CA (2017). Prevalence of metabolic syndrome among apparently healthy adults in Ogun state. Nigeria.

[48] Owolabi EO, Ter Goon D, Adeniyi OV, Adedokun AO, Seekoe E (2017). Prevalence and correlates of metabolic syndrome among adults attending healthcare facilities in Eastern Cape, South Africa. Open Public Health J..

[49] Zhao Y, Yan H, Yang R, Li Q, Dang S, Wang Y (2014). Prevalence and determinants of metabolic syndrome among adults in a rural area of northwest China. PLoS One.

[50] De Carvalho Vidigal F, Ribeiro AQ, Babio N, Salas-Salvadó J, Bressan J (2015). Prevalence of metabolic syndrome and pre-metabolic syndrome in health professionals: LATINMETS Brazil study. Diabetol. Metab. Syndr..

[51] Kaur J (2014). Assessment and screening of the risk factors in metabolic syndrome. Med. Sci..

[52] Salminen A, Ojala J, Kaarniranta K, Kauppinen A (2012). Mitochondrial dysfunction and oxidative stress activate inflammasomes: Impact on the aging process and age-related diseases. Cell. Mol. Life Sci..

[53] Laakso M, Kuusisto J (2014). Insulin resistance and hyperglycaemia in cardiovascular disease development. Nat. Rev. Endocrinol..

[54] Wagner A (2012). Sedentary behaviour, physical activity and dietary patterns are independently associated with the metabolic syndrome. Diabetes Metab..

[55] González-Muniesa P (2017). Obesity. Nat. Rev. Dis. Prim..

[56] Adamczak M, Wiecek A (2013). The adipose tissue as an endocrine organ. Semin. Nephrol..

[57] Bosho DD, Dube L, Mega TA, Adare DA, Tesfaye MG, Eshetie TC (2018). Prevalence and predictors of metabolic syndrome among people living with human immunodeficiency virus (PLWHIV). Diabetol. Metab. Syndr..

[58] Tesfaye DY (2014). Burden of metabolic syndrome among HIV-infected patients in Southern Ethiopia. Diabetes Metab. Syndr. Clin. Res. Rev..

[59] Salas R (2014). Metabolic syndrome prevalence among Northern Mexican adult population. PLoS One.

[60] Review AS, Banik SD (2018). Prevalence of metabolic syndrome in Mexico. Metab. Syndrome Relat. Disord..

[61] Mabry RM, Reeves MM, Eakin EG, Owen N (2010). Short report gender differences in prevalence of the metabolic syndrome in Gulf cooperation Council Countries: A systematic review. Diab. Med..

[62] Ntandou G, Delisle H, Agueh V, Fayomi B (2009). Abdominal obesity explains the positive rural-urban gradient in the prevalence of the metabolic syndrome in Benin, West Africa. Nutr. Res..

